# Detailed operational building data for six office rooms in Denmark: Occupancy, indoor environment, heating, ventilation, lighting and room control monitoring with sub-hourly temporal resolution

**DOI:** 10.1016/j.dib.2024.110326

**Published:** 2024-03-16

**Authors:** Simon Pommerencke Melgaard, Hicham Johra, Victor Ørsøe Nyborg, Anna Marszal-Pomianowska, Rasmus Lund Jensen, Christos Kantas, Olena Kalyanova Larsen, Yue Hu, Kirstine Meyer Frandsen, Tine Steen Larsen, Kjeld Svidt, Kamilla Heimar Andersen, Daniel Leiria, Markus Schaffer, Martin Frandsen, Martin Veit, Lene Faber Ussing, Søren Munch Lindhard, Michal Zbigniew Pomianowski, Lasse Rohde, Anders Rhiger Hansen, Per Kvols Heiselberg

**Affiliations:** a Department of the Built Environment, Aalborg University, Thomas Manns vej 23, 9220 Aalborg Øst, Denmark; b Department of Architecture, Design and Media Technology, Aalborg University, Rendsburggade 14, 9000 Aalborg, Denmark

**Keywords:** High-resolution, Indoor climate, Building systems, Office building, Number of occupants, HVAC

## Abstract

The operational building data presented in this paper has been collected from six office rooms located in an office building (research and educational purposes) located on the main campus of Aalborg University in Denmark. The dataset consists of measurements of occupancy, indoor environmental quality, room-level and system-level heating, ventilation and lighting operation at a 5 min resolution. The indoor environmental quality and building system data were collected from the building management system. The occupancy level in each monitored room is established from the computer vision-based analysis of wall-mounted camera footage of each office. The number of people present in the room is estimated using the *YOLOv5s* image recognition algorithm. The present dataset can be used for occupancy analysis, indoor environmental quality investigations, machine learning, and model predictive control.

Specifications Table

**Table:** 

Subject	Architecture, Control and Systems Engineering
Specific subject area	Building systems, indoor climate measurements, room control, occupancy monitoring
Data format	Analyzed (building management system data converted to 5 min resolution) Analyzed (room overview images converted to number of occupants with 5 min resolution)
Type of data	.csv file (dataset with building management system data and number of occupants) .xlsx file (dataset with building management system data and number of occupants) .py file (data visualization)
Data collection	The indoor environmental quality and building system data was collected from the sensors permanently mounted in the building through the building management system (BMS). The occupancy data was generated from images taken by wall-mounted cameras in the monitored rooms and analyzed *via* the *YOLOv5* algorithm to count the number of occupants. The data was collected from the 27^th^ of February, 2023 until the 31^st^ of December, 2023.
Data source location	Six offices, one air handling unit, and one heating system located at Thomas Manns vej 23, 9220 Aalborg Øst, Denmark. The building belongs to the Department of the Built Environment at Aalborg University. Coordinates: 57°00’52.2”N 9°58’23.5”E
Data accessibility	Repository name: zenodo.org Data identification name: *A high-resolution dataset for six office rooms in Denmark with occupancy, indoor environment, heating, ventilation, lighting and room control monitoring.* Data identification number: 10.5281/zenodo.10039896 Direct URL to data: https://doi.org/10.5281/zenodo.10039896

## 1 Value of the Data

• The dataset has a high time resolution of five minutes and spans almost a full year (including three different seasons, with both heating and cooling periods), which is currently rare in the building sector.
• The dataset covers most room-level control and indoor environmental variables typically found in building management systems (BMS) of office buildings, along with all control and measurement values for the connected heating, ventilation, and air conditioning (HVAC) central systems connected to the six monitored rooms. Room-level artificial lighting activation and presence detection from passive infrared (PIR) sensors are also included.
• The ground truth on the occupancy of the rooms (number of people present in each room at a given time) is accurately established from computer vision-based analysis of camera footage from each monitored room. This information is very rarely present in building datasets.
• Besides the dataset, a detailed description of each room and the building systems is provided, thus leaving no missing information for most building applications.
• Researchers focusing on occupant detection through BMS data, building indoor environmental analysis, air handling unit (AHU) performance analysis, and model predictive control (MPC) could benefit from this dataset due to its high resolution and completeness.

## 2 Data Description

The dataset is comprised of one full dataset (dataset__2023_02_27__2023_12_31) and four subsets covering a winter case (dataset__2023_03_08__2023_03_21), a winter/transition period case (dataset__2023_04_01__2023_04_13), a summer case (dataset__2023_06_01__2023_07_05) and a summer/transition period case (dataset__2023_09_02__2023_10_04).

Only the full dataset is presented here.

The datasets are available in two file types: either .xlsx or .csv. All .xlsx files contain a metadata sheet with all the data variables’ descriptions and the corresponding number of missing data points in each file. The .csv files only contain the dataset. The .csv file uses a semicolon as separator between columns (variables) and a period as decimal separator. Missing values are indicated by #N/A. Both file types contain a starting index in the first column, which can be used to easily find where the subsets are positioned in the full dataset. The first row in both file types is the header, with the naming of each variable explained in the following subsection.

### 2.1 Dataset__2023_02_27__2023_12_31

This dataset [1] comprises the following parts:

- Room-level indoor environmental quality, presence detection from PIR sensor, and occupancy measurements, along with artificial lighting, radiator valve, and variable air volume (VAV) damper operational data in six different office rooms.
  ○ These always start with the label “RoomX:” where X is a letter from A to F.
- Measurements of the central AHU connected to the six office rooms.
  ○ These always start with the label “Ventilation:”
  ○ One should note that the AHU supplies more than just the six rooms of this dataset.
- Measurements of the central heating system supplying the radiator to the six office rooms.
  ○ These always start with the label “Heating:”
  ○ One should note that the central heating system supplies more than just the six rooms of this dataset.
- Measurements of the outdoor conditions.
  ○ These always start with the label “Outdoor:”

The timestamp in the file is in the format “YYYY-MM-DDThh:mm:ss+hhmm” according to ISO 8601 and is showing the local Danish time (time zone Europe/Copenhagen), which in standard time is UTC+1 (CET) and daylight-saving time is UTC+2 (CEST). Transitions between standard time and daylight-saving time were on March 26th, 2023, at 02:00 (CET) and back to standard time on October 29th, 2023, at 03:00 (CEST).

Table 2, Table 3, Table 4, Table 5, Table 6, Table 7, Table 8, Table 9, Table 10, Table 11 contain all the variables for each room, system and outdoor condition. The “Limits on operating range” column indicates any natural limits for the respective variables. The following options can be found:

- “-“ means it is unrestricted.
- “0-“ means it cannot be lower than 0.
- “0/1” means it is a Boolean, such as on or off.
- “0-1” means an averaged Boolean value, such as on or off, but it can be any decimal value between 0 and 1 due to data treatment and averaging, thus indicating the share of state 1 between the current and previous timestamp.
- “0-100” means it can be any integer between 0 and 100, but due to data treatment and averaging, it can become any decimal value between 0 and 100.
- “1/10/14” means that only these specific values can occur, but due to data treatment and averaging, decimal values other than these can occur when transitioning between the values.

#### 2.1.1 Room-Level Measurements

**Fig. 1:**
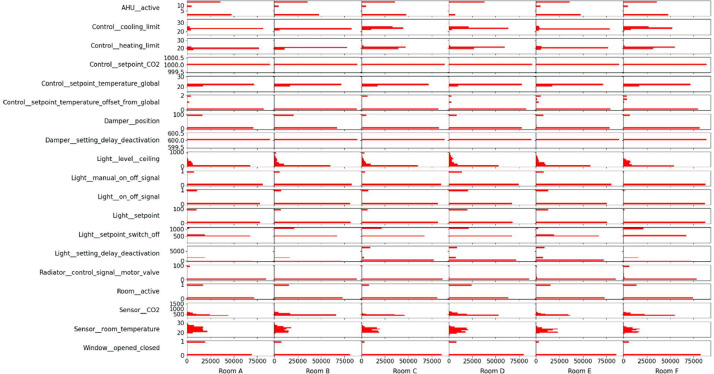
Room measurements’ distribution over the entire monitoring period.

**Fig. 2:**
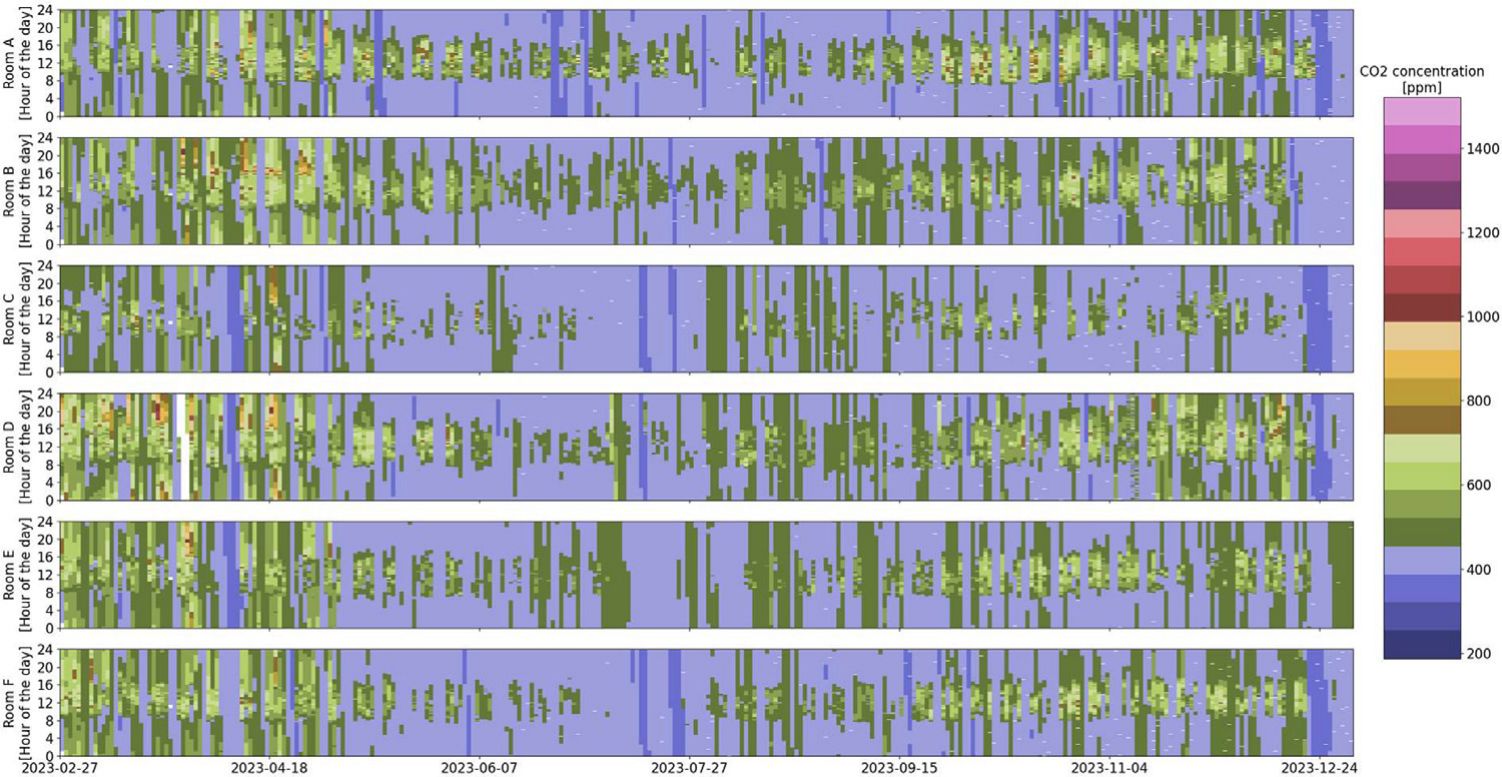
CO_2_ concentration overview in the six rooms over the entire monitoring period (white indicates missing data).

**Fig. 3:**
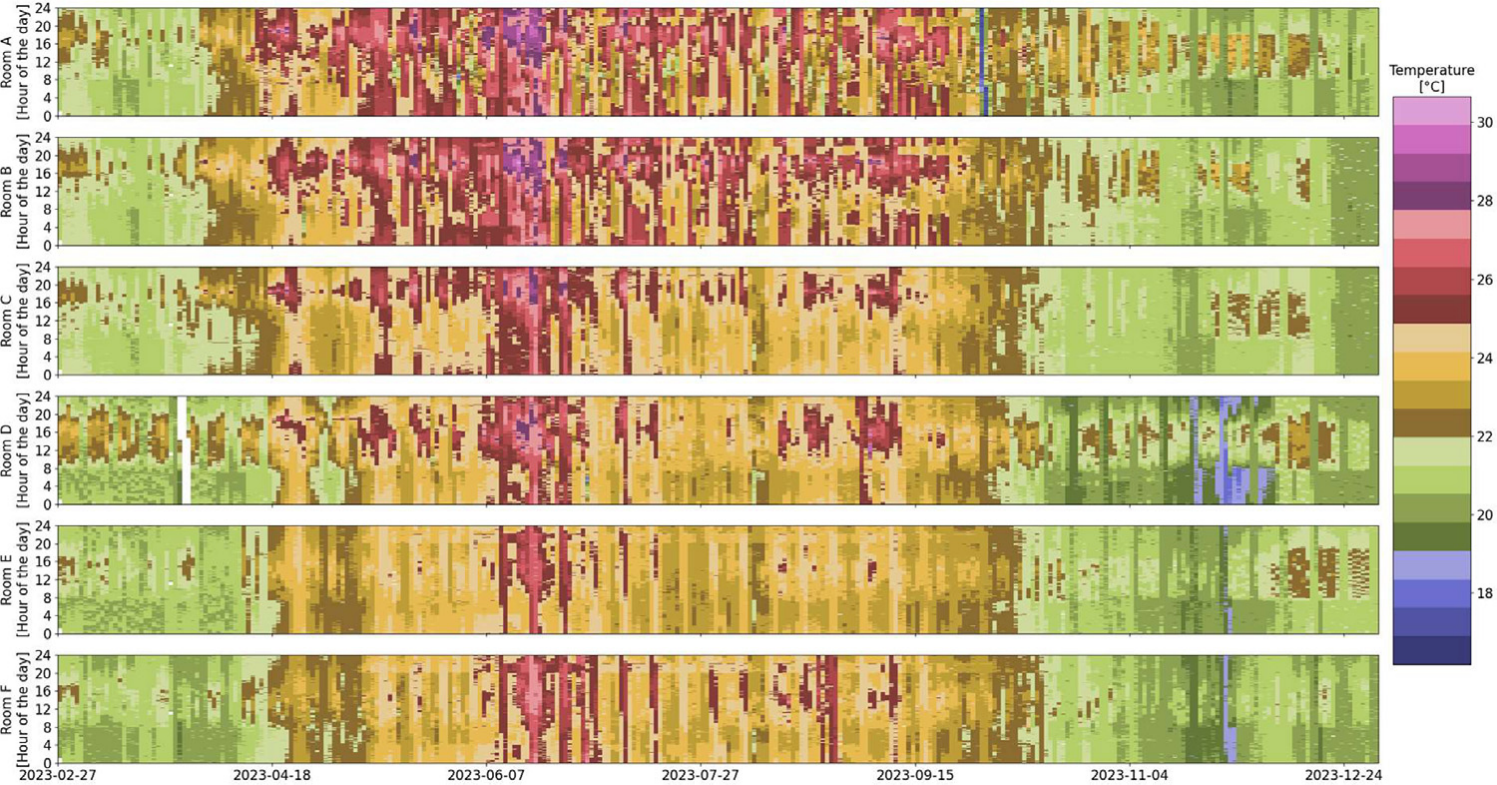
Room temperature overview in the six rooms over the entire monitoring period (white indicates missing data).

**Fig. 4:**
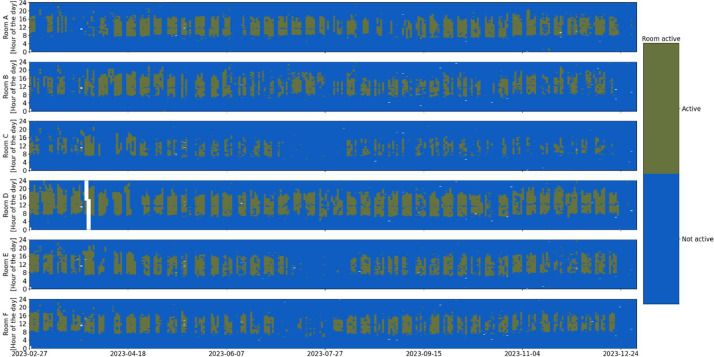
Room presence detection (PIR sensor) overview in the six rooms over the entire monitoring period (white indicates missing data).

**Fig. 5:**
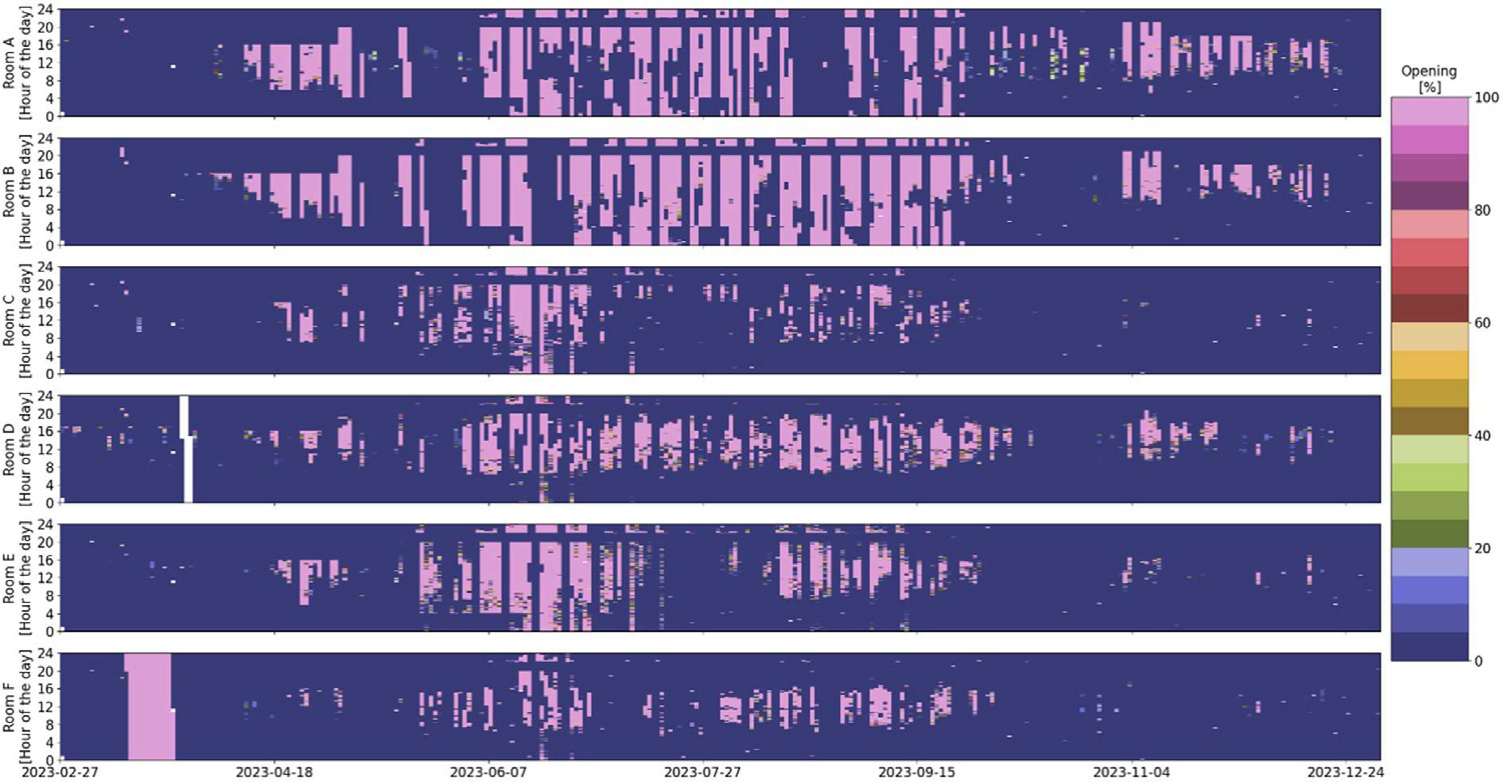
VAV damper position overview in the six rooms over the entire monitoring period. 0% opening means that the damper is at minimum presetting of the damper; see Table 1 for more information (white indicates missing data).

**Fig. 6:**
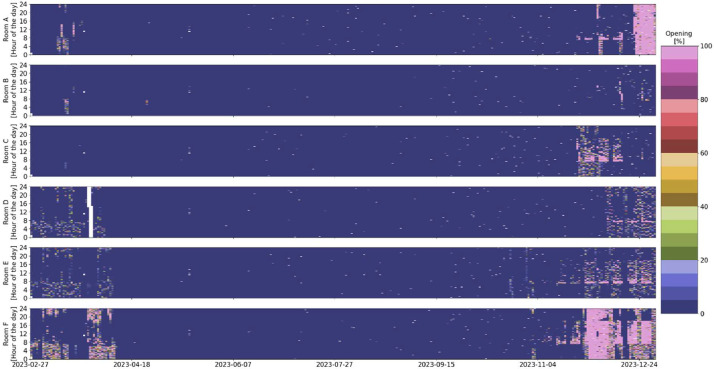
Radiator valve position overview in the six rooms over the entire monitoring period (white indicates missing data).

**Fig. 7:**
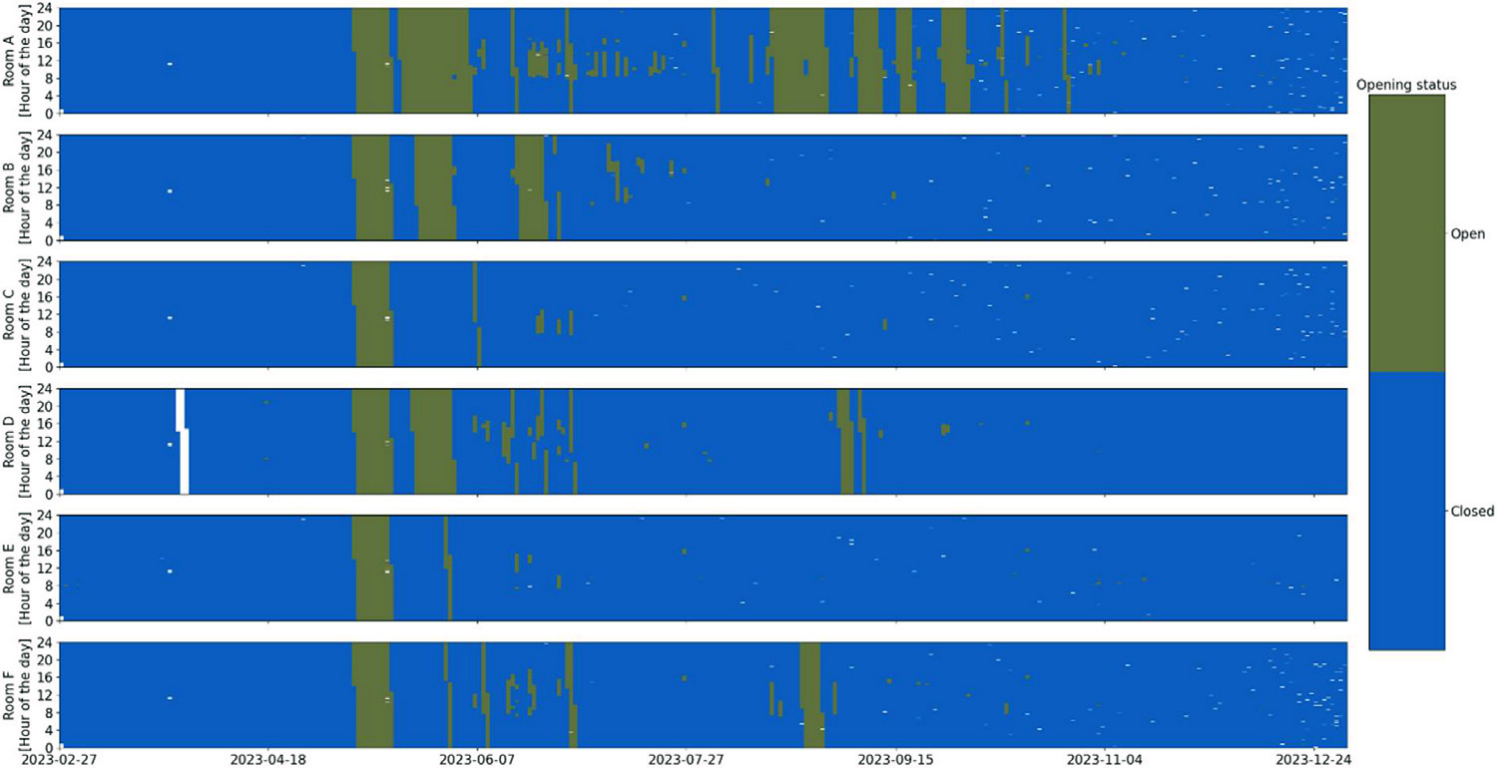
Window opening overview in the six rooms over the entire monitoring period (white indicates missing data).

An overview of the room-level measurement variables available for each room, along with a short description of the meaning of each variable, can be found in Table 1. The distribution overview of all the different room measurements can be seen in Fig. 1. Some room measurement visualizations in Fig. 2, Fig. 3, Fig. 4, Fig. 5, Fig. 6, Fig. 7 were generated using the Python script in Ref. [1].

**Table 1:** General overview of data available for the rooms: X indicates that the measurement is available in the room.

Variable	Meaning of variable	Found in Room A	Found in Room B	Found in Room C	Found in Room D	Found in Room E	Found in Room F
RoomX:AHU__active	Status of the supplying AHU. (1 means the AHU is off. 10 means it is in night ventilation mode. 14 means it is in normal operation mode)	X	X	X	X	X	X
RoomX:Control__cooling_limit	The upper limit of the temperature deadband. (used for opening/closing the VAV damper due to temperature)	X	X	X	X	X	X
RoomX:Control__deadband_temperature_day	Deadband applied during normal day operation mode. (added on both sides of the sum of global temperature setpoint (all rooms in the building) and local temperature setpoint offset (room-specific))			X	X	X	X
RoomX:Control__deadband_temperature_day_standby	Deadband applied during standby day operation mode. (added on both sides of the sum of global temperature setpoint and local temperature setpoint offset)			X	X	X	X
RoomX:Control__deadband_temperature_night	Deadband applied during night operation mode. (added on both sides of the sum of global temperature setpoint and local temperature setpoint offset)			X	X	X	X
RoomX:Control__heating_limit	The lower limit of the temperature deadband. (used for opening/closing the radiator valve)	X	X	X	X	X	X
RoomX:Control__setpoint_CO2	The upper limit for CO2 concentration in the room. (used for opening/closing the VAV damper)	X	X	X	X	X	X
RoomX:Control__setpoint_temperature_global	The global building temperature setpoint. (set globally for all rooms in the building)	X	X	X	X	X	X
RoomX:Control__setpoint_temperature_offset_from_global	The local temperature offset in the room. (used to deviate from the global building temperature setpoint and can be up to ±2.5 °C)	X	X	X	X	X	X
RoomX:Damper__position	Position of both the supply and extraction VAV dampers in the room. (0% opening means that the dampers are at the minimum presetting, roughly 30% of maximum airflow). The measured relationship between opening and airflow can be seen in the table below. Be aware that this relation could change due to pressure differences in the system.	X	X	X	X	X	X
RoomX:Damper__setting_delay_deactivation	How long the VAV damper stay open after activity (PIR sensor activation) has stopped in the room.	X	X	X	X	X	X
RoomX:Light__level__ceiling	The light level measured at the ceiling of the room.	X	X	X	X	X	X
RoomX:Light__manual_on_off_signal	Manually turning the light switch on/off.	X	X	X	X	X	X
RoomX:Light__on_off_signal	Actual on/off signal for the light.	X	X	X	X	X	X
RoomX:Light__setpoint	Set point for the light as a percentage of the maximum lighting illuminance. (if it is above 0, the light is turned on).	X	X	X	X	X	X
RoomX:Light__setpoint_switch_off	The upper limit for the light. (in relation to the Light__level__ceiling, the light is turned off if the Light__level__ceiling exceeds this threshold)	X	X	X	X	X	X
RoomX:Light__setting_delay_deactivation	The delay between no activity in the room (PIR sensor activation) and turning off the light.	X	X	X	X	X	X
RoomX:Radiator__control_signal__motor_valve	Opening percentage of the motor valve for all the radiators in the room.	X	X	X	X	X	X
RoomX:Room__active	If the room is considered active due to the presence of people (has a delay of 10 min from the last PIR sensor signal before being considered inactive).	X	X	X	X	X	X
RoomX:Sensor__CO2	Measurements from the room's control panel CO2 sensor.	X	X	X	X	X	X
RoomX:Sensor__room_temperature	Measurements from the room's control panel temperature sensor.	X	X	X	X	X	X
RoomX:Window__opened_closed	If 0, all windows are closed. If 1, at least one window is open. (if the window is open, the motor valves for the radiators and the VAV dampers are closed by the control).	X	X	X	X	X	X

**Table 2:** Room A measurement variables.

Variable	Unit	Limits on operating range	Number of data points	Number of missing data points	Missing data points in percentage
RoomA:AHU__active	–	1/10/14	88,479	226	0.3%
RoomA:Control__cooling_limit	°C	–	88,641	64	0.1%
RoomA:Control__heating_limit	°C	–	88,639	66	0.1%
RoomA:Control__setpoint_CO2	ppm	0-	88,687	18	0.0%
RoomA:Control__setpoint_temperature_global	°C	–	88,652	53	0.1%
RoomA:Control__setpoint_temperature_offset_from_global	°C	–	88,444	261	0.3%
RoomA:Damper__position	%	0-100	88,648	57	0.1%
RoomA:Damper__setting_delay_deactivation	s	0-	88,647	58	0.1%
RoomA:Light__level__ceiling	lux	0-	88,642	63	0.1%
RoomA:Light__manual_on_off_signal	–	0/1	88,647	58	0.1%
RoomA:Light__on_off_signal	–	0/1	88,464	241	0.3%
RoomA:Light__setpoint	%	0–100	88,529	176	0.2%
RoomA:Light__setpoint_switch_off	lux	0-	88,649	56	0.1%
RoomA:Light__setting_delay_deactivation	s	0-	88,644	61	0.1%
RoomA:Radiator__control_signal__motor_valve	%	0–100	88,472	233	0.3%
RoomA:Room__active	–	0/1	88,652	53	0.1%
RoomA:Sensor__CO2	ppm	0-	88,468	237	0.3%
RoomA:Sensor__room_temperature	°C	–	88,660	45	0.1%
RoomA:Window__opened_closed	–	0/1	88,535	170	0.2%

**Table 3:** Room B measurement variables.

Variable	Unit	Limits on operating range	Number of data points	Number of missing data points	Missing data points inpercentage
RoomB:AHU__active	–	1/10/14	88,476	229	0.3%
RoomB:Control__cooling_limit	°C	–	88,516	189	0.2%
RoomB:Control__heating_limit	°C	–	88,639	66	0.1%
RoomB:Control__setpoint_CO2	ppm	0-	88,687	18	0.0%
RoomB:Control__setpoint_temperature_global	°C	–	88,649	56	0.1%
RoomB:Control__setpoint_temperature_offset_from_global	°C	–	88,435	270	0.3%
RoomB:Damper__position	%	0–100	88,647	58	0.1%
RoomB:Damper__setting_delay_deactivation	s	0-	88,651	54	0.1%
RoomB:Light__level__ceiling	lux	0-	88,646	59	0.1%
RoomB:Light__manual_on_off_signal	–	0/1	88,649	56	0.1%
RoomB:Light__on_off_signal	–	0/1	88,485	220	0.2%
RoomB:Light__setpoint	%	0–100	88,530	175	0.2%
RoomB:Light__setpoint_switch_off	lux	0-	88,652	53	0.1%
RoomB:Light__setting_delay_deactivation	s	0-	88,640	65	0.1%
RoomB:Radiator__control_signal__motor_valve	%	0–100	88,512	193	0.2%
RoomB:Room__active	–	0/1	88,657	48	0.1%
RoomB:Sensor__CO2	ppm	0-	88,472	233	0.3%
RoomB:Sensor__room_temperature	°C	–	88,425	280	0.3%
RoomB:Window__opened_closed	–	0/1	88,551	154	0.2%

**Table 4:** Room C measurement variables.

Variable	Unit	Limits on operating range	Number of data points	Number of missing data points	Missing data points in percentage
RoomC:AHU__active	–	1/10/14	88,491	214	0.2%
RoomC:Control__cooling_limit	°C	–	88,506	199	0.2%
RoomC:Control__deadband_temperature_day	°C	–	88,445	260	0.3%
RoomC:Control__deadband_temperature_day_standby	°C	–	88,507	198	0.2%
RoomC:Control__deadband_temperature_night	°C	–	88,658	47	0.1%
RoomC:Control__heating_limit	°C	–	88,643	62	0.1%
RoomC:Control__setpoint_CO2	ppm	0-	88,687	18	0.0%
RoomC:Control__setpoint_temperature_global	°C	–	88,646	59	0.1%
RoomC:Control__setpoint_temperature_offset_from_global	°C	–	88,466	239	0.3%
RoomC:Damper__position	%	0–100	88,642	63	0.1%
RoomC:Damper__setting_delay_deactivation	s	0-	88,643	62	0.1%
RoomC:Light__level__ceiling	lux	0-	88,637	68	0.1%
RoomC:Light__manual_on_off_signal	–	0/1	88,646	59	0.1%
RoomC:Light__on_off_signal	–	0/1	88,472	233	0.3%
RoomC:Light__setpoint	%	0–100	88,530	175	0.2%
RoomC:Light__setpoint_switch_off	lux	0-	88,645	60	0.1%
RoomC:Light__setting_delay_deactivation	S	0-	88,637	68	0.1%
RoomC:Radiator__control_signal__motor_valve	%	0–100	88,479	226	0.3%
RoomC:Room__active	–	0/1	88,654	51	0.1%
RoomC:Sensor__CO2	ppm	0-	88,475	230	0.3%
RoomC:Sensor__room_temperature	°C	–	88,654	51	0.1%
RoomC:Window__opened_closed	–	0/1	88,550	155	0.2%

**Table 5:** Room D measurement variables.

Variable	Unit	Limits on operating range	Number of data points	Number of missing data points	Missing data points in percentage
RoomD:AHU__active	–	1/10/14	44,856	43,849	49.4%
RoomD:Control__cooling_limit	°C	–	87,980	725	0.8%
RoomD:Control__deadband_temperature_day	°C	–	87,911	794	0.9%
RoomD:Control__deadband_temperature_day_standby	°C	–	88,103	602	0.7%
RoomD:Control__deadband_temperature_night	°C	–	88,103	602	0.7%
RoomD:Control__heating_limit	°C	–	88,058	647	0.7%
RoomD:Control__setpoint_CO2	ppm	0-	88,687	18	0.0%
RoomD:Control__setpoint_temperature_global	°C	–	88,102	603	0.7%
RoomD:Control__setpoint_temperature_offset_from_global	°C	–	88,102	603	0.7%
RoomD:Damper__position	%	0–100	88,103	602	0.7%
RoomD:Damper__setting_delay_deactivation	s	0-	88,101	604	0.7%
RoomD:Light__level__ceiling	lux	0-	87,980	725	0.8%
RoomD:Light__manual_on_off_signal	–	0/1	88,064	641	0.7%
RoomD:Light__on_off_signal	–	0/1	87,951	754	0.9%
RoomD:Light__setpoint	%	0–100	88,007	698	0.8%
RoomD:Light__setpoint_switch_off	lux	0-	88,008	697	0.8%
RoomD:Light__setting_delay_deactivation	S	0-	88,062	643	0.7%
RoomD:Radiator__control_signal__motor_valve	%	0–100	87,966	739	0.8%
RoomD:Room__active	–	0/1	88,068	637	0.7%
RoomD:Sensor__CO2	ppm	0-	87,940	765	0.9%
RoomD:Sensor__room_temperature	°C	–	88,071	634	0.7%
RoomD:Window__opened_closed	–	0/1	88,103	602	0.7%

**Table 6:** Room E measurement variables.

Variable	Unit	Limits on operating range	Number of data points	Number of missing data points	Missing data points in percentage
RoomE:AHU__active	–	1/10/14	88,484	221	0.2%
RoomE:Control__cooling_limit	°C	–	88,510	195	0.2%
RoomE:Control__deadband_temperature_day	°C	–	88,422	283	0.3%
RoomE:Control__deadband_temperature_day_standby	°C	–	88,640	65	0.1%
RoomE:Control__deadband_temperature_night	°C	–	88,657	48	0.1%
RoomE:Control__heating_limit	°C	–	88,636	69	0.1%
RoomE:Control__setpoint_CO2	ppm	0-	88,687	18	0.0%
RoomE:Control__setpoint_temperature_global	°C	–	88,645	60	0.1%
RoomE:Control__setpoint_temperature_offset_from_global	°C	–	88,451	254	0.3%
RoomE:Damper__position	%	0–100	88,641	64	0.1%
RoomE:Damper__setting_delay_deactivation	s	0-	88,654	51	0.1%
RoomE:Light__level__ceiling	lux	0-	88,633	72	0.1%
RoomE:Light__manual_on_off_signal	–	0/1	88,642	63	0.1%
RoomE:Light__on_off_signal	–	0/1	88,490	215	0.2%
RoomE:Light__setpoint	%	0–100	88,534	171	0.2%
RoomE:Light__setpoint_switch_off	lux	0-	88,644	61	0.1%
RoomE:Light__setting_delay_deactivation	s	0-	88,643	62	0.1%
RoomE:Radiator__control_signal__motor_valve	%	0–100	88,483	222	0.3%
RoomE:Room__active	–	0/1	88,651	54	0.1%
RoomE:Sensor__CO2	ppm	0-	88,647	58	0.1%
RoomE:Sensor__room_temperature	°C	–	88,658	47	0.1%
RoomE:Window__opened_closed	–	0/1	88,635	70	0.1%

**Table 7:** Room F measurement variables.

Variable	Unit	Limits on operating range	Number of data points	Number of missing data points	Missing data points in percentage
RoomF:AHU__active	–	1/10/14	88,523	182	0.2%
RoomF:Control__cooling_limit	°C	–	88,519	186	0.2%
RoomF:Control__deadband_temperature_day	°C	–	88,430	275	0.3%
RoomF:Control__deadband_temperature_day_standby	°C	–	88,527	178	0.2%
RoomF:Control__deadband_temperature_night	°C	–	88,652	53	0.1%
RoomF:Control__heating_limit	°C	–	88,641	64	0.1%
RoomF:Control__setpoint_CO2	ppm	0-	88,687	18	0.0%
RoomF:Control__setpoint_temperature_global	°C	–	88,635	70	0.1%
RoomF:Control__setpoint_temperature_offset_from_global	°C	–	88,448	257	0.3%
RoomF:Damper__position	%	0–100	88,638	67	0.1%
RoomF:Damper__setting_delay_deactivation	s	0-	88,640	65	0.1%
RoomF:Light__level__ceiling	lux	0-	88,627	78	0.1%
RoomF:Light__manual_on_off_signal	–	0/1	88,628	77	0.1%
RoomF:Light__on_off_signal	–	0/1	88,499	206	0.2%
RoomF:Light__setpoint	%	0–100	88,547	158	0.2%
RoomF:Light__setpoint_switch_off	lux	0-	88,648	57	0.1%
RoomF:Light__setting_delay_deactivation	s	0-	88,628	77	0.1%
RoomF:Radiator__control_signal__motor_valve	%	0–100	88,502	203	0.2%
RoomF:Room__active	–	0/1	88,650	55	0.1%
RoomF:Sensor__CO2	ppm	0-	88,476	229	0.3%
RoomF:Sensor__room_temperature	°C	–	88,660	45	0.1%
RoomF:Window__opened_closed	–	0/1	88,562	143	0.2%

**Table 8:** Occupancy variables for the six rooms.

Variable	Unit	Limits on operating range	Comment	Number of data points	Number of missing data points	Missing data points in percentage
RoomA:People__amount	–	0-	Number of people present in the room.	48,902	39,803	44.9%
RoomB:People__amount	–	0-	Number of people present in the room.	40,892	47,813	53.9%
RoomC:People__amount	–	0-	Number of people present in the room.	58,848	29,857	33.7%
RoomD:People__amount	–	0-	Number of people present in the room.	53,569	35,136	39.6%
RoomE:People__amount	–	0-	Number of people present in the room.	49,278	39,427	44.4%
RoomF:People__amount	–	0-	Number of people present in the room.	50,454	38,251	43.1%

**Table 9:** AHU measurement variables.

Variable	Unit	Limits on operating range	Comment	Number of data points	Number of missing data points	Missing data points in percentage
Ventilation:AHU__SEL	J/m^3^	0-	The SFP value of the AHU. (This value includes all the electricity used by the entire AHU).	88,687	18	0.0%
Ventilation:AHU__active	-	1/10/14	Current operation mode for the AHU. (1=the AHU is turned off, 10=the AHU is running in night operation mode, 14=the AHU is in normal day operation mode).	88,687	18	0.0%
Ventilation:Control__setpoint_air_pressure__extraction	Pa	0-	Pressure to be kept in the extraction duct.	88,687	18	0.0%
Ventilation:Control__setpoint_air_pressure__supply	Pa	0-	Pressure to be kept in the supply duct.	88,687	18	0.0%
Ventilation:Control__setpoint_air_temperature__supply	°C	–	Air temperature to be kept in the supply duct.	88,687	18	0.0%
Ventilation:Damper__on_off_signal__extraction	–	0–1	Shut off damper position on the extraction side. (0=closed, 1=open)	88,687	18	0.0%
Ventilation:Damper__on_off_signal__intake	–	0–1	Shut off damper position on the intake side. (0=closed, 1=open)	88,687	18	0.0%
Ventilation:EL_meter__energy_accumulated	kWh	0-	Total electricity use of the AHU.	88,687	18	0.0%
Ventilation:EL_meter__power	W	0-	Electrical power of the entire AHU.	88,687	18	0.0%
Ventilation:Fan__air_flow__supply	m^3^/h	0-	Airflow across the supply fan. In the period 2023-02-27T00:00:00+0100 – 2023-07-13T08:00:00+0200 the flow has been corrected by lowering it by 1800 m^3^/h.	88,687	18	0.0%
Ventilation:Fan__control_signal__exhaust	%	0–100	Modulating control signal for the exhaust fan.	88,687	18	0.0%
Ventilation:Fan__control_signal__supply	%	0–100	Modulating control signal for the supply fan.	88,687	18	0.0%
Ventilation:Fan__on_off_signal__exhaust	–	0–1	On or off signal for the exhaust fan. (0=off, 1=on).	88,687	18	0.0%
Ventilation:Fan__on_off_signal__supply	–	0–1	On or off signal for the supply fan. (0=off, 1=on).	88,687	18	0.0%
Ventilation:Fan__power__exhaust	kW	0-	Power drawn by the exhaust fan.	88,687	18	0.0%
Ventilation:Fan__power__supply	kW	0-	Power drawn by the supply fan.	88,687	18	0.0%
Ventilation:Fan__pressure_difference__supply	Pa	0-	Pressure difference across the supply fan. In the period 2023-02-27T00:00:00+0100 – 2023-07-13T08:00:00+0200 the pressure difference has been corrected by calculating it based on the flow and the fans k-factor of 308.	88,687	18	0.0%
Ventilation:HC__DH_temperature__return	°C	–	District heating return temperature on the heating coil.	88,687	18	0.0%
Ventilation:HC__DH_temperature__supply	°C	–	District heating supply temperature on the heating coil.	88,687	18	0.0%
Ventilation:HC__control_signal__mixing_valve	%	0–100	Heating coil signal for mixing return water with DH supply water.	88,687	18	0.0%
Ventilation:HC__control_water_temperature_minimum_limit__return	°C	–	Minimum temperature for the return on the heating coil to prevent freezing and condensation problems.	88,687	18	0.0%
Ventilation:HC__energy_accumulated	Wh	0-	Energy use of the heating coil.	88,687	18	0.0%
Ventilation:HC__on_off_signal__pump	–	0–1	On or off signal for the heating coil pump. (0=off, 1=on).	88,687	18	0.0%
Ventilation:HC__operation_hours_accumulated	h	0-	Number of hours the heating coil has been in operation.	88,687	18	0.0%
Ventilation:HC__power	W	0-	Current thermal power drawn by the heating coil.	88,687	18	0.0%
Ventilation:HC__water_flow	l/s	0-	Current district heating water flow drawn by the heating coil.	88,687	18	0.0%
Ventilation:HC__water_flow_accumulated	l	0-	Accumulated district heating water drawn by the heating coil.	88,687	18	0.0%
Ventilation:HC__water_temperature__return	°C	–	Temperature directly after the heating coil.	88,687	18	0.0%
Ventilation:HC__water_temperature__supply	°C	–	Temperature directly before the heating coil.	88,687	18	0.0%
Ventilation:HE__efficiency	%	0–100	Heat exchanger heat recovery efficiency. (Due to the calculation method in the BMS system, the calculation of the heat recovery efficiency of the rotary heat exchanger is only valid for balanced flow)	88,687	18	0.0%
Ventilation:HE__rotation_signal	%	0–100	Rotation signal for the heat exchanger.	88,687	18	0.0%
Ventilation :Sensor__air_pressure__extraction	Pa	0-	Air pressure measured in the extraction duct.	88,687	18	0.0%
Ventilation :Sensor__air_pressure__supply	Pa	0-	Air pressure measured in the supply duct.	88,687	18	0.0%
Ventilation:Sensor__air_temperature__after_HE_before_HC	°C	–	Air temperature measured between the heat exchanger and the heating coil.	88,687	18	0.0%
Ventilation:Sensor__air_temperature__exhaust	°C	–	Air temperature measured in the exhaust duct.	88,687	18	0.0%
Ventilation :Sensor__air_temperature__extraction	°C	–	Air temperature measured in the extraction duct.	88,687	18	0.0%
Ventilation:Sensor__air_temperature__intake	°C	–	Air temperature measured in the intake duct.	88,687	18	0.0%
Ventilation:Sensor__air_temperature__supply	°C	–	Air temperature measured in the supply duct.	88,687	18	0.0%

**Table 10:** Heating system measurement variables.

Variable	Unit	Limits on operating range	Comment	Number of data points	Number of missing data points	Missing data points in percentage
Heating:Control__setpoint_water_temperature__supply	°C	0-	Setpoint for the supply temperature.	88,687	18	0.0%
Heating:DH__energy_accumulated	Wh	0-	Accumulated energy use of the district heating.	88,687	18	0.0%
Heating:DH__operation_hours_accumulated	h	0-	Accumulated number of operational hours.	88,687	18	0.0%
Heating:DH__power	W	0-	Current power drawn from the district heating.	88,687	18	0.0%
Heating:DH__water_flow	l/s	0-	Current water flow drawn from the district heating.	88,687	18	0.0%
Heating:DH__water_flow_accumulated	l	0-	Accumulated district heating water drawn.	88,687	18	0.0%
Heating:DH__water_temperature__return	°C	–	Return temperature of the district heating.	88,687	18	0.0%
Heating:DH__water_temperature__supply	°C	–	Supply temperature of the district heating.	88,687	18	0.0%
Heating:Mixing_valve__control_signal	%	0–100	Control signal for the mixing valve for keeping the supply temperature setpoint.	88,687	18	0.0%
Heating:Pump__on_off_signal	–	0–1	On or off signal for the pump. (0=off, 1=on).	88,687	18	0.0%
Heating:Pump__setting_outdoor_temperature_activate	°C	–	Outdoor temperature below which the pump is started.	88,687	18	0.0%
Heating:Pump__setting_outdoor_temperature_deactivate	°C	–	Outdoor temperature above which the pump is stopped.	88,687	18	0.0%
Heating:Sensor__water_temperature__return	°C	–	Return temperature measured before the mixing valve.	88,687	18	0.0%
Heating:Sensor__water_temperature__supply	°C	–	Supply temperature measured after mixing.	88,687	18	0.0%

#### 2.1.2 Occupancy Measurements

The real-time occupancy for each room is the current number of occupants detected in that room at a given time. The six rooms have the following number of desks (potential fixed working spaces for occupants):

- Room A: 5 desks
- Room B: 4 desks
- Room C: 3 desks
- Room D: 6 desks
- Room E: 4 desks
- Room F: 4 desks

**Fig. 8:**
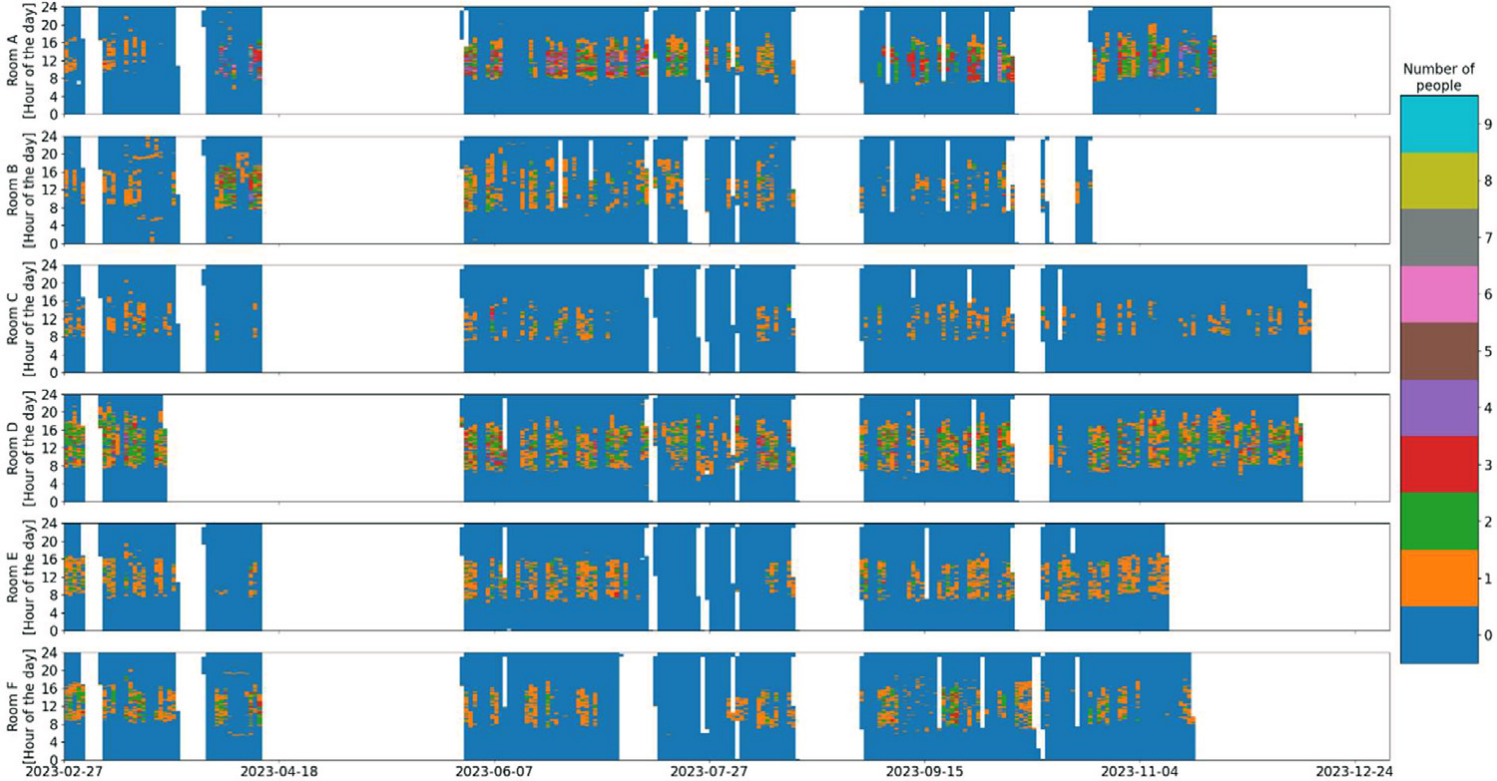
Occupancy overview in the six rooms over the entire monitoring period (white indicates missing data).

**Fig. 9:**
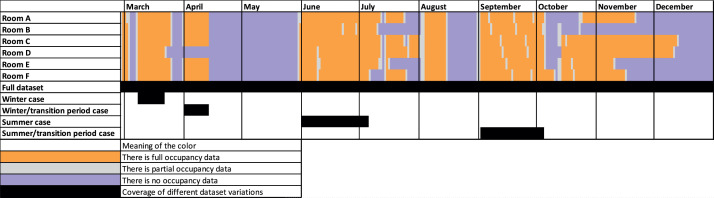
Overview of available data on occupancy from camera footage of the six office rooms.

The actual number of occupants over the measurement period can be seen in Fig. 8, while the periods where the occupancy data is available are shown in Fig. 9.

#### 2.1.3 Air Handling Unit measurements

An overview of the variables can be seen in Table 9.

#### 2.1.4 Heating System Measurements

An overview of the variables can be seen in Table 10.

#### 2.1.5 Outdoor Measurements

**Fig. 10:**
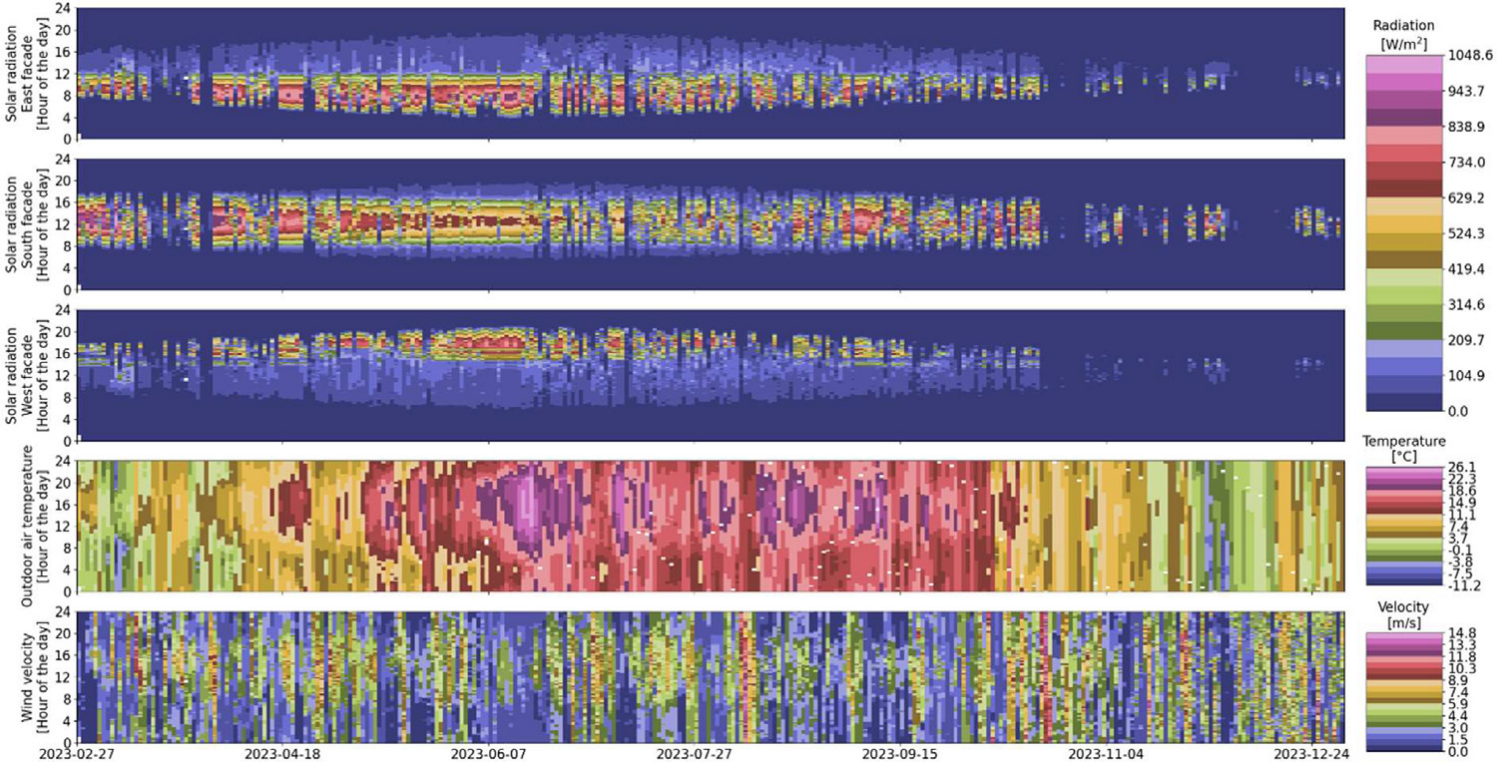
Outdoor conditions overview over the entire monitoring period (white indicates missing data).

An overview and visualization of the variables can be seen in Fig. 10 and Table 11.

**Table 11:** Outdoor measurement variables.

Variable	Unit	Limits on operating range	Comment	Number of data points	Number of missing data points	Missing data points in percentage
Outdoor:Solar__direct_radiation__east_façade	W/m^2^	0-	Direct solar radiation measured on the east façade of the building.	88,687	18	0.0%
Outdoor:Solar__direct_radiation__south_façade	W/m^2^	0-	Direct solar radiation measured on the south façade of the building.	88,687	18	0.0%
Outdoor:Solar__direct_radiation__west_façade	W/m^2^	0-	Direct solar radiation measured on the west façade of the building.	88,687	18	0.0%
Outdoor:Temperature_air	°C	-	Outdoor air temperature measured at the building rooftop. (Measured every 15 min and linearly interpolated to get 5 min values)	88,292	413	0.5%
Outdoor:Wind__velocity	m/s	0-	Measured at the building rooftop.	88,687	18	0.0%

## 3 Experimental Design, Materials and Methods

A general description of the case study building can be found in Johra, 2023 [2].

All the data, except for the occupancy, was collected from the BMS and either resampled or realigned to a 5 min resolution. For the BMS data, all measurements from the rooms originally had a sampling rate of five minutes and were therefore only realigned by shifting the logged timestamp to the aligned timestamp, this was done as the logged timestamp was within 1 min of the aligned timestamp, thus it was deemed close enough. The HVAC and outdoor measurements (except for the outdoor temperature) originally had a sampling rate of one minute and, therefore, were downsampled to 5 min resolution using the mean value. This process will remove extremes to some degree, but as the fluctuation in most cases is low, it will only have a minor impact. Any missing data has been labelled as #N/A, as no imputation of data was performed. “Outdoor:Temperature_air” was linearly interpolated from 15 min values to 5 min values. In the case when one of the 15 min values was missing, no interpolation was done between this point and its neighboring datapoints.

The supply airflow and pressure across the supply fan were recalculated in the period between 2023-02-27T00:00:00+0100 and 2023-06-13T08:00:00+0200, as an improper connection in the sensor was found to cause too high pressure difference and thus too high flow measurements (details of the correction can be seen in Table 9).

The number of occupants in each room at a given time was determined by analyzing the footage of a wall-mounted camera installed in each monitored room with a computer vision-based algorithm that accurately detects humans. An image was taken every one minute during extended work hours (07:00 – 18:00 during standard time (before 2023-03-26T02:00:00+0100 and after 2023-10-29T02:00:00+0100), otherwise 08:00 – 19:00 during daylight savings time). Outside of the work hours the pictures were only taken every five minutes. All the images were processed using a pre-trained *YOLOv5s* algorithm with default settings [3] to identify the number of occupants in each image. The accuracy of the prediciton model is discussed in the Limitations section. The images were aligned with the BMS data by using the image with the closest timestamp for each BMS data point. If no images were found within ± 10 min of the BMS datapoint, the image was regarded as missing, and an #N/A value was recorded for the occupancy level measurement of this data point.

**Fig. 11:**
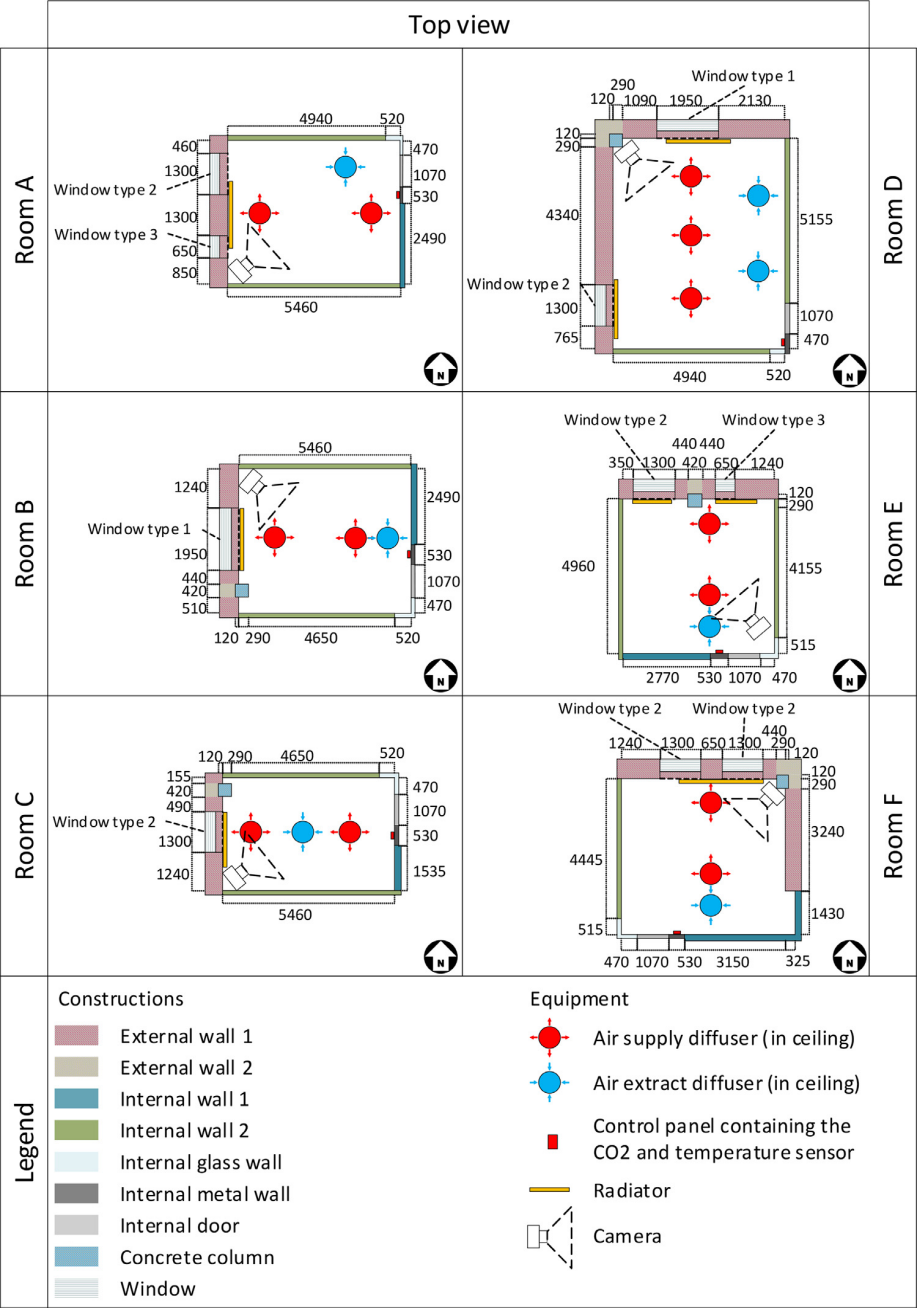
Schematic of six office rooms (with geometry, window types, wall types, building systems and sensors). All dimensions are in mm.

**Fig. 12:**
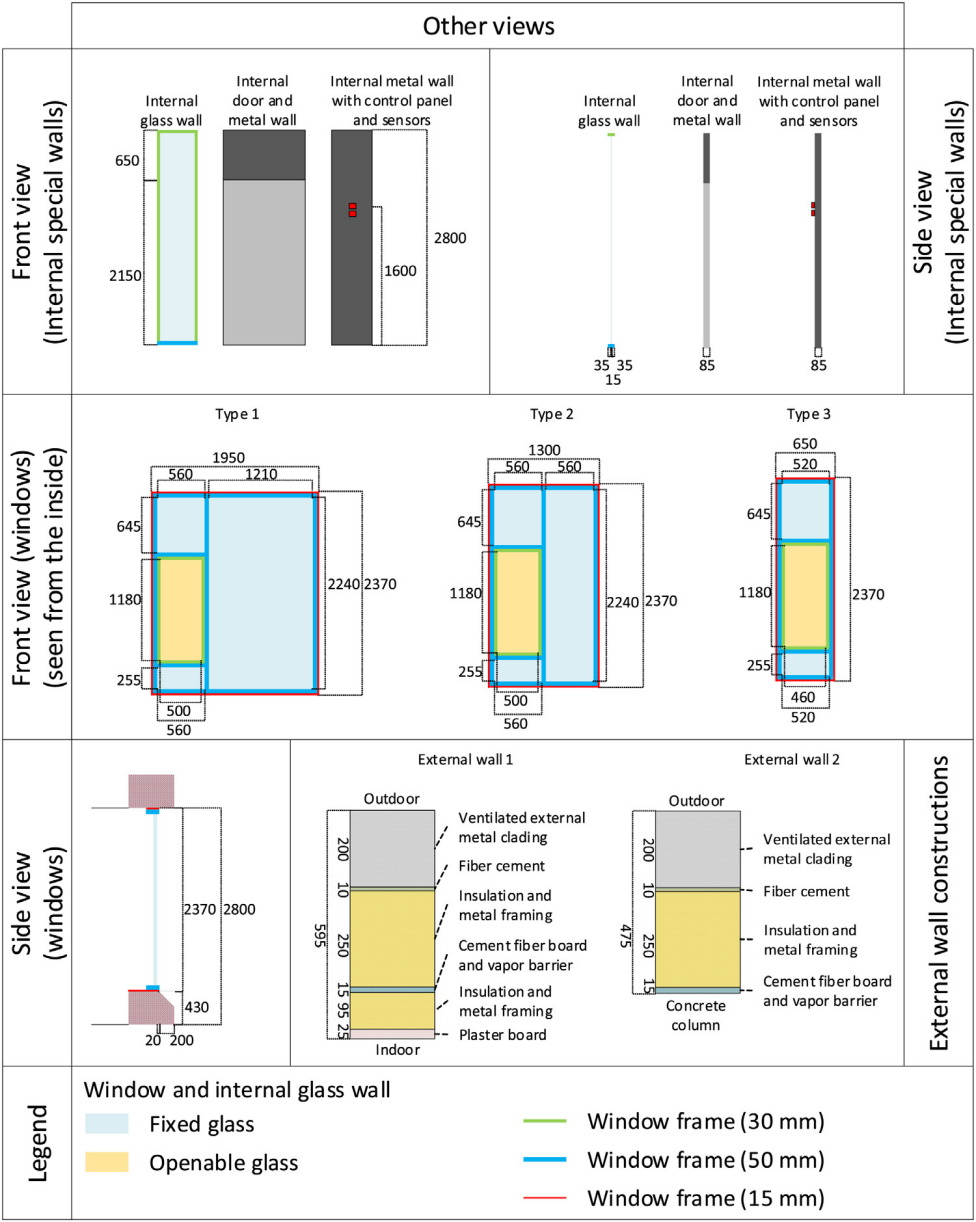
Schematic of the wall and window types. All dimensions are in mm.

**Fig. 13:**
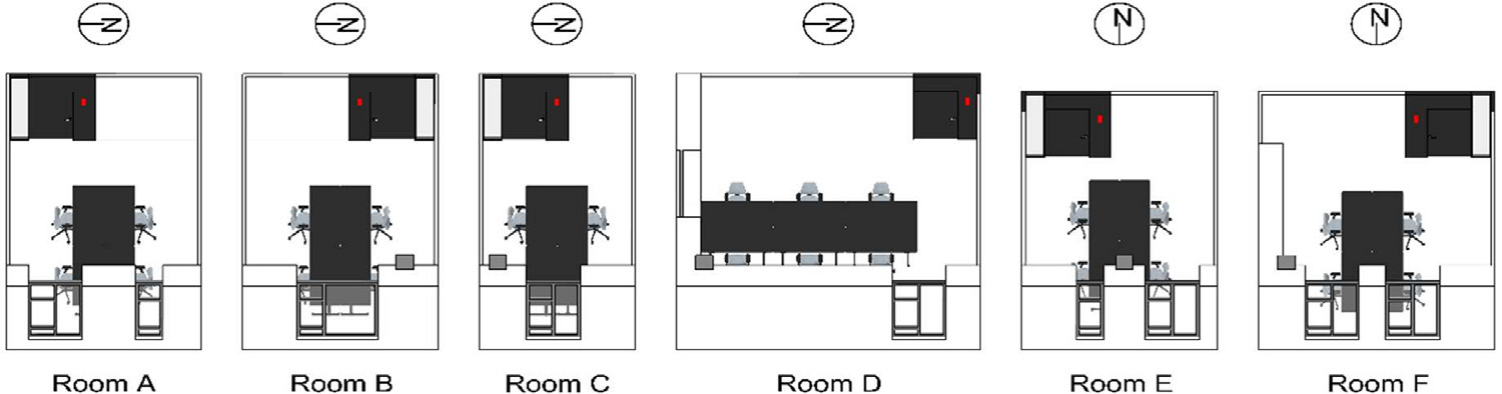
3D visualization of the rooms including the position of the fixed working spaces (office chairs) and the location of the temperature and CO_2_ sensor in each room (red square near the door).

**Fig. 14:**
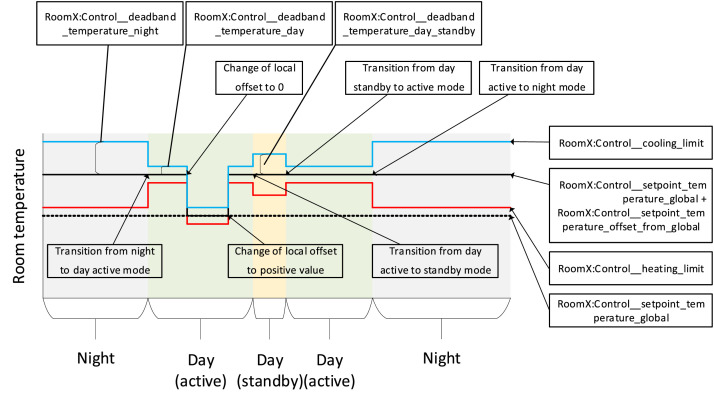
Heating and cooling control states and limits for the rooms.

**Fig. 15:**
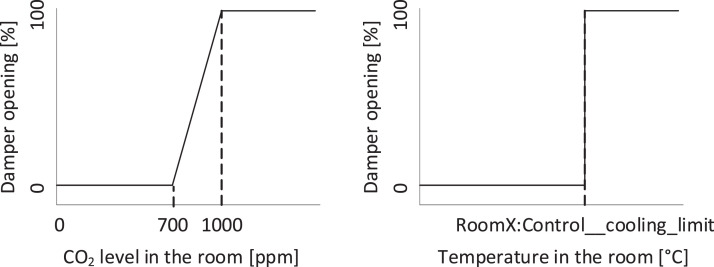
Temperature and CO_2_ setpoint curve for the VAV damper. A damper opening of 0% corresponds to an airflow of 30% of the maximum airflow.

All rooms are equipped with VAV dampers for the ventilation distribution system, along with radiators for the heating system. The overall schematic of the rooms can be seen in Figs. 11, 12, 13. The rooms all have balanced ventilation, with the VAV dampers in each room being controlled by the same signal for supply and extraction. It is important to note that 0% opening of the dampers corresponds to roughly 30% of the maximum airflow to ensure the base minimum ventilation rate. The measured relationship between damper opening and airflow rate for each room can be seen in Table 1. When a room window is opened, the VAV dampers and the radiator control valve are turned to 0%. The dampers and heating system in each room are controlled with a PI controller according to a temperature setpoint with a deadband. This deadband varies depending on the room and time of day. It can be found directly for rooms C-F while it must be calculated from the heating and cooling limits for rooms A-B. An illustration of the relationship between control variables used for the heating and cooling control can be seen in Fig. 14. The opening of the damper depends on the highest value of the opening signal for temperature, and CO_2_ setpoint. The characteristic of the temperature and CO_2_ opening signals can be seen in Fig. 15.

**Fig. 16:**
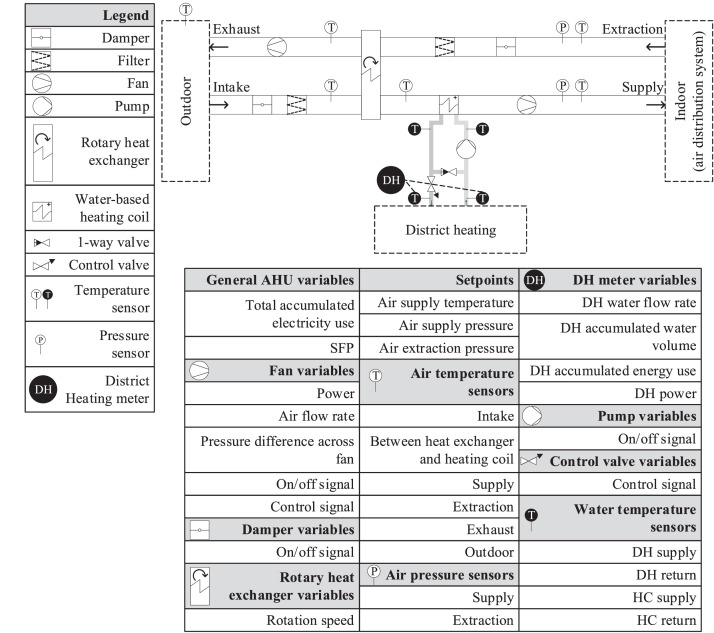
Schematic for the AHU, along with an overview of the sensors providing data to the BMS.

The AHU is a VAV unit with a rotating wheel heat recovery unit and a water-based heating coil supplied by district heating (DH). During normal operation, the AHU is controlled to maintain a specific temperature setpoint for the supply air, and a specific air pressure setpoint in both the supply and extraction ducts. All controllers are PI-controllers with a small deadband of either 0.1 °C or 1 Pa. The AHU supplies air to one seminar room, two meeting rooms, 21 offices, six toilets, eight auxiliary rooms, and hallways/open areas on three floors. A schematic of the AHU and the locations of the measurement points can be seen in Fig. 16.

**Fig. 17:**
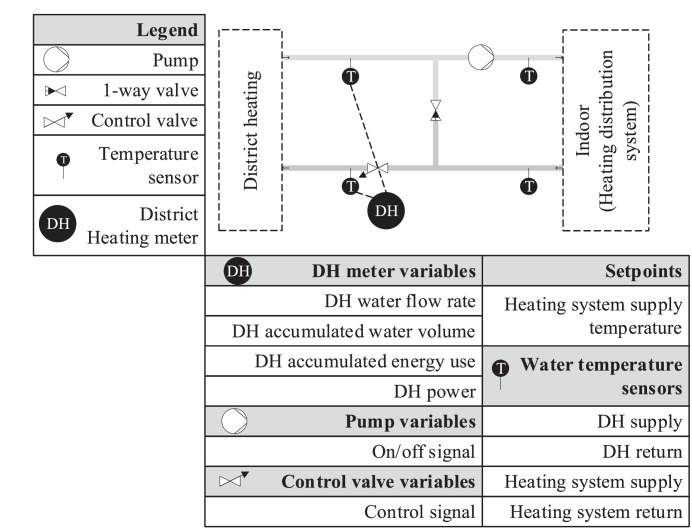
Schematic for the heating system, along with an overview of the sensors providing data to the BMS.

The heating system provides heat for roughly half of the building. It is a direct DH-based system with a mixing control, meaning that depending on the heating need of the building, the return water from the building will be recirculated and mixed with the DH water supply to ensure that the temperature setpoint of the supply to the building is met. The mixing ratio is controlled by the control valve located on the DH return, as the pump is only controlled with an on/off controller. A schematic of the heating system can be seen in Fig. 17.

The outdoor measurements come from sensors located on the building's rooftop.

The sensors used in the rooms and systems, along with their accuracy has been summarized in Table 12.

**Table 12:** Sensors and their information for the different measurement points.

Related to variable	Sensor name and model	Measurement information	Other information
RoomX:Light__level__ceiling	Philips LRI8134/00 SENSR MULTISENSOR		
RoomX:Room__active			
RoomX:Sensor__CO2	Schneider Electric SCR110	Measurement range: 0–2000 ppm Accuracy: ±1.5% of measurement range Accuracy: ±2% of measured value Repeatability: ±20 ppm ±1% of measured value	Sensor Type: Non-dispersive infrared (NDIR), diffusion sampling
RoomX:Sensor__room_temperature		Measurement range: 0–50 °C Accuracy: ±0.5 °C	Sensor Type: Thermistor
Ventilation:EL_meter__power	Schneider Electric Acti9 iEM3175		
Ventilation:Fan__power__exhaust	Schneider Electric ATV212HU22N4	Accuracy: ±0.6% VIA ±0.6% VIB ±1% FM All for a temperature variation 60 °C	VIA is the primary speed reference VIB is the sceondary speed reference FM is the output frequency
Ventilation:Fan__power__supply			
Ventilation:Fan__pressure_difference__exhaust	Schneider Electric EPP302LCD	Measurement range: 0–2500 Pa Accuracy: ±1% of measurement range	Used to calculate the corresponding airflow using the fan k-factor of 308 and the formula: flow = k * sqrt(pres_diff)
Ventilation:Fan__pressure_difference__supply			
Ventilation:HC__DH_temperature__return	Kamstrup MULTICAL 602C02480A1545+ Kamstrup ULTRAFLOW 1465-1-CDAA-545	Temperature range: 2–50 °C Differential range: 3–40 K Accuracy: ±(0.5+DR_min_/DR_value_)%	
Ventilation:HC__DH_temperature__supply			
Ventilation:HC__power		Calculator accuracy: ±(0.15+2/DR_value_)% Sensor set accuracy: ±(0.4+4/DR_value_)% Flow sensor accuracy: ±(1+0.01*nominal flow/ flow)%	DR_value_ refers to the differential value from temperatures
Ventilation:HC__water_flow		Nominal flow: 1.5 m^3^/h	
Ventilation:HC__water_temperature__return	Schneider Electric STP100-100	Accuracy at -25 °C: ±0.7 °C 0 °C: ±0.5 °C 25 °C: ±0.3 °C 50 °C: ±0.6 °C 75 °C: ±0.9 °C	
Ventilation:HC__water_temperature__supply			
Ventilation:Sensor__air_pressure__extraction	Schneider Electric SPD310-100…1000 Pa	Measurement range: 0–300 Pa Accuracy: >100 Pa is ≤ 0.75% of measurement range <100 Pa is ≤ 1.5% of measurement range	
Ventilation:Sensor__air_pressure__supply			
Ventilation:Sensor__air_temperature__after_HE_before_HC	Schneider Electric STD100-300	Accuracy at -25 °C: ±0.7 °C 0 °C: ±0.5 °C 25 °C: ±0.3 °C 50 °C: ±0.6 °C 75 °C: ±0.9 °C	
Ventilation:Sensor__air_temperature__exhaust			
Ventilation:Sensor__air_temperature__extraction			
Ventilation:Sensor__air_temperature__intake			
Ventilation:Sensor__air_temperature__supply			
Heating:DH__power	Kamstrup MULTICAL 602C02480A1245 + Kamstrup ULTRAFLOW 14 65-1-CGAG-545	Calculator accuracy: ±(0.15+2/DR_value_)% Sensor set accuracy: ±(0.4+4/DR_value_)% Flow sensor accuracy: ±(1+0.01*nominal flow/ flow)%	DR_value_ refers to the differential value from temperatures
Heating:DH__water_temperature__return		Temperature range: 2–180 °C Differential range: 3–170 K Accuracy: ±(0.5+DR_min_/DR_value_)%	
Heating:DH__water_temperature__supply			
Heating:DH__water_flow		Nominal flow: 3.5 m^3^/h	
Heating:Sensor__water_temperature__return Heating:Sensor__water_temperature__supply	Schneider Electric STP100-100	Accuracy at -25 °C: ±0.7 °C 0 °C: ±0.5 °C 25 °C: ±0.3 °C 50 °C: ±0.6 °C 75 °C: ±0.9 °C	

## Limitations

Due to practical limitations on the possible locations of the cameras in each room, some areas were difficult to detect people in, as the image did not capture the entire person. To mitigate this issue, the cameras were placed to cover all the fixed working stations and the entrance door of the rooms. Manual performance verification of the people detection algorithm (checking randomly picked-up images and labeled occupancy) shows that the number of people in the room is correctly determined in more than 99% of the cases (26 false occupancy assessments out of 5188 image samples). On rare occasions, people outside the room were also detected when passing in front of the open door or visible through the glazed surface next to the door.

## Ethics Statement

To conduct the experiments for the generation of the present dataset, the appropriate administrative body (Aalborg University – AAU Innovation – Grants & Contracts) has been contacted in order to verify the ethical soundness of the experiment and the necessary measures that had to be taken regarding the General Data Protection Regulation (GDPR). After informing this administrative body (Aalborg University – AAU Innovation – Grants & Contracts), all the participants in the experiment have been informed about the use of the collected data and a GDPR consent form has been sent to them. The authors hereby confirm that the relevant, informed consent was obtained from all subjects who have participated in the generation of that dataset. A copy of the original consent form can be found in the appendix. Copies of the signed informed consent are retained by the authors. No additional approval from institutional review boards or local ethics committees was necessary to conduct this experiment.

The current dataset and the present dataset description do not comprise any personal or specific information, which could lead to the identification of the subjects who have participated in the generation of that dataset.

## CRediT authorship contribution statement

**Simon Pommerencke Melgaard:** Conceptualization, Methodology, Software, Investigation, Data curation, Writing – original draft, Writing – review & editing, Visualization. **Hicham Johra:** Conceptualization, Methodology, Software, Investigation, Writing – original draft, Writing – review & editing, Funding acquisition. **Victor Ørsøe Nyborg:** Software, Investigation. **Anna Marszal-Pomianowska:** Methodology, Resources. **Rasmus Lund Jensen:** Supervision, Resources. **Christos Kantas:** Software. **Olena Kalyanova Larsen:** Resources. **Yue Hu:** Resources. **Kirstine Meyer Frandsen:** Resources. **Tine Steen Larsen:** Resources. **Kjeld Svidt:** Resources. **Kamilla Heimar Andersen:** Resources. **Daniel Leiria:** Resources. **Markus Schaffer:** Writing – review & editing, Visualization, Resources. **Martin Frandsen:** Resources. **Martin Veit:** Resources. **Lene Faber Ussing:** Resources. **Søren Munch Lindhard:** Resources. **Michal Zbigniew Pomianowski:** Resources. **Lasse Rohde:** Resources. **Anders Rhiger Hansen:** Resources. **Per Kvols Heiselberg:** Supervision, Resources, Funding acquisition.

## Data Availability

A Danish high-resolution dataset for six office rooms with occupancy, indoor environment , heating, ventilation, lighting and room control monitoring (Original data) (Zenodo).
